# Problems with Facial Mimicry Might Contribute to Emotion Recognition Impairment in Parkinson's Disease

**DOI:** 10.1155/2018/5741941

**Published:** 2018-11-11

**Authors:** Margaret T. M. Prenger, Penny A. MacDonald

**Affiliations:** ^1^Brain and Mind Institute, Western University, London, ON, Canada; ^2^Clinical Neurological Sciences, Schulich School of Medicine & Dentistry, Western University, London, ON, Canada

## Abstract

Difficulty with emotion recognition is increasingly being recognized as a symptom of Parkinson's disease. Most research into this area contends that progressive cognitive decline accompanying the disease is to be blamed. However, facial mimicry (i.e., the involuntary congruent activation of facial expression muscles upon viewing a particular facial expression) might also play a role and has been relatively understudied in this clinical population. In healthy participants, facial mimicry has been shown to improve recognition of observed emotions, a phenomenon described by embodied simulation theory. Due to motor disturbances, Parkinson's disease patients frequently show reduced emotional expressiveness, which translates into reduced mimicry. Therefore, it is likely that facial mimicry problems in Parkinson's disease contribute at least partly to the emotional recognition deficits that these patients experience and might greatly influence their social cognition abilities and quality of life. The present review aims to highlight the need for further inquiry into the motor mechanisms behind emotional recognition in Parkinson's disease by synthesizing behavioural, physiological, and neuroanatomical evidence.

## 1. Introduction

Parkinson's disease (PD) is a neurodegenerative illness arising due to the loss of dopamine-producing neurons in the substantia nigra pars compacta (SNc) [1, 2]. The loss of these neurons leads to a reduction in dopamine neurotransmission in the basal ganglia, resulting in overinhibition of certain motor and cognitive pathways [3]. The basal ganglia are subcortical nuclei comprising the striatum, globus pallidus, substantia nigra, and subthalamic nucleus [4, 5]. Common motor symptoms include reduced number and amplitude of movements (i.e., hypokinesia), slowed movement (i.e., bradykinesia), rigidity, tremor, and instability, whereas cognitive symptoms include impaired working memory, attention, and cognitive flexibility [1–3]. Emotional processing impairments are increasingly acknowledged by clinicians and researchers as another common symptom of PD [6, 7].

Recent reviews on emotion processing in PD patients have highlighted that patients display a global reduction in emotion recognition abilities, with specific deficits for negative emotions such as disgust [6–9]. However, deficits have been found for all six basic emotions (happiness, surprise, anger, sadness, fear, and disgust) across a number of studies [10–12]. This impairment has been shown to be independent of general deficits in face processing [13], emotion regulation abilities [14, 15], problems with lower-order vision [16], and cognitive function [17]. However, there appears to be a large degree of inconsistency within the literature, as there are several factors that make it difficult to compare results across studies (e.g., medication status, length/stage of illness, and depression). Consequently, it is difficult to determine the cause or contributing factors behind emotion recognition deficits.

Another common observation in PD is reduced emotional expressivity, which has been demonstrated for both spontaneous and voluntary displays of emotion. This is thought to arise due to dopamine depletion in the basal ganglia leading to hypokinesia and bradykinesia of the facial muscles [18]. Interestingly, some studies have shown there exists a relationship between reductions in emotional expressivity and emotion recognition [8, 10, 15].

This link between emotional recognition and expressivity might be explained by an important mirroring behaviour. Facial mimicry describes the unconscious mirroring of others' emotional facial expressions by activating one's own congruent facial muscles [19]. Embodied simulation theory suggests that facial mimicry aids in the recognition and understanding of others' emotions through an emotional feedback mechanism. Once an expression is mimicked, the brain associates the accompanying sensorimotor feedback signals with a certain emotion, which is then experienced on some level by the observer and recognized [20, 21]. Indeed, healthy participants have shown reductions in emotion recognition abilities when facial mimicry is blocked [22–24]. Neuroimaging research has also elucidated areas of the brain that are highly active during facial mimicry, particularly in areas of the basal ganglia (e.g., caudate nucleus) and of the mirror neuron system (e.g., inferior frontal operculum) [25, 26].

Given that emotional expressivity [15, 27], basal ganglia function [28], and mirror neuron system activity [29] are all reduced in PD patients, it is likely that facial mimicry is also reduced. However, there are currently only two studies that have investigated facial mimicry in PD patients [30, 31]. Based on the demonstrated link between facial mimicry and emotional recognition, it seems likely that PD patients' reductions in facial mimicry contribute at least somewhat to their impairment in emotion recognition. The present review therefore aims to summarize evidence surrounding emotional recognition and expressivity in PD patients, facial mimicry in healthy participants and PD patients, and the neural correlates of facial mimicry and emotion recognition. The overarching aim of this review is to underline the need for further inquiry in the area of facial mimicry in PD patients. We predict that facial mimicry is an important, relatively unexplored contributing factor to emotion recognition impairments in PD patients.

## 2. Emotion Recognition in PD

Recent reviews on emotion recognition in PD patients have highlighted a large degree of inconsistency within the literature on this topic. While most reviews agree that emotion recognition deficits are found in the majority of studies, there are many contradictions and incidental findings. According to Assogna et al. [7], disgust is the facial expression which is least recognized by patients with PD. For example, using an assessment which controlled task difficulty, Suzuki et al. [32] identified a specific impairment for disgust recognition in dopamine-medicated PD patients compared to controls. Interestingly, this result was corroborated by Sprengelmeyer and colleagues [33], but only for PD patients off medication; those that were on medication failed to show any difference in the recognition of disgust compared to controls.

In their review, Argaud et al. [6] reported that 64% of relevant studies found a global reduction in the recognition of the six basic emotions. However, the authors also highlight a multitude of factors which may confound results, including a lack of stimuli continuity, varying levels of task difficulty, and failure to control for dopaminergic therapy. For example, static facial expression photographs are widely used as stimuli in emotion recognition research, but dynamic video stimuli tend to be more ecologically valid [34]. Argaud and colleagues explain that the use of static stimuli might artificially inflate the emotion recognition deficit in PD patients, as dynamic emotional stimuli have indeed been found to be more easily recognized by PD patients [6, 35].

In their meta-analysis, Gray and Tickle-Degnen [8] also observed a general deficit in emotion recognition for PD patients compared to healthy controls. In addition, they suggest that the recognition of negative emotions is more impaired than that of positive emotions. Indeed, Lawrence et al. [36] found that PD patients off of their normal dopamine therapy regime had difficulty recognizing anger. However, Aiello and colleagues [37] also tested patients off medication and found no difference from controls in anger recognition (or for any emotions, for that matter). Taken together, these reviews demonstrate that emotion recognition abilities do seem to be reduced in PD patients but that the literature is not entirely clear on how or why. As a final note, many studies investigating emotion recognition in PD patients controlled for potential confounding factors such as bradykinesia which would slow response times [13, 38] and depression [39].

## 3. Facial Expressiveness in PD Patients

Facial hypomimia, or “masking” of facial expression, is a symptom of PD arising from bilateral hypokinesia and bradykinesia of the muscles of facial expression [18]. As a result, PD patients are less able to produce emotional expressions and can appear “cold” or unhappy to others. Spontaneous emotional expressions seem to be particularly affected by PD, as these are mediated by a habitual control loop between the basal ganglia and the cortex which is especially damaged by the disease [18,40–43]. For example, Simons et al. [44] made PD patients and healthy controls watch amusing videos designed to elicit spontaneous expressions. During the videos, PD patients were significantly less expressive than controls. This was not due to a decreased ability to feel emotions, as both PD patients and controls indicated a similar level of self-reported amusement during these videos. Similarly, Ricciardi et al. [15] documented reduced emotional expressivity in PD patients compared to controls who were asked to describe a typical day.

Simons and colleagues [27] cleverly investigated the interplay between spontaneous and voluntary facial expression in PD patients by having participants pose incongruent expressions while smelling certain odours (e.g., posing a smile during a foul odour). Compared to controls, PD patients demonstrated a reduced ability to mask their negative expressions with a positive one, with many patients showing blending of both positive and negative expressions. This result intriguingly suggests that deficits in facial expression might not simply result from hypokinesia and bradykinesia leading to general facial muscular control problems. Simons et al.'s [27] findings are analogous to the cognitive control deficits that occur in PD patients. It is well documented that PD patients have difficulty averting attention from more naturally salient to less salient stimuli or overriding more automatic or innate responses when they are incongruous with requested but less habitual responses [45–50]. In this way, subtle cognitive changes associated with PD could also contribute to facial expression deficits.

### 3.1. Relationship between Emotion Recognition and Emotional Expression in PD Patients

Deficits in emotion recognition and expression for PD patients seem to be related, as evidenced by correlations in several studies. For example, Ricciardi and colleagues [10] made participants to complete the Ekman 60 Faces test (a test of emotional recognition) and imitate the expressions that were displayed. Compared to controls, PD patients performed worse at recognizing all of the six basic emotions (except for disgust) and were judged to be less expressive for happiness, anger, and sadness, which was accompanied by a correlation of *r* = 0.48 between the ability to express and recognize the six basic emotions. In fact, an even greater correlation was found when the emotions were ranked from best to least recognized (*r* = 0.75). Similarly, in their meta-analysis, Gray and Tickle-Dengen [8] report that the average correlation between recognition and expressivity across a number of studies was *r* = 0.47. It is important to note that not all studies have demonstrated this link, however. Although Bologna and colleagues [51] found deficits in emotional expressivity and emotional recognition in PD patients, there was no correlation between these two deficits. Correlation does not imply causation, making these studies in PD patients difficult to interpret. There is evidence in healthy populations; however, that interfering with emotional expression reduces emotion recognition as will be detailed in the sections below.

## 4. Facial Mimicry

Facial mimicry is a nonconscious, involuntary behaviour involving congruent activation of facial muscles upon observing another's expression [19]. For example, when observing a happy face, one tends to activate the *zygomaticus major* muscles, which turn the corners of the mouth upwards into a smile, as well as the *orbicularis oculi* muscles, which are responsible for squinting the eyes. Similarly, when observing an angry face, one tends to activate the *corrugator supercilii* muscles, which furrow the brow [19]. This behaviour occurs very quickly (within 300–400 msec) and is present from infancy [52, 53].

Research on facial mimicry has shown that it might be related to processing the information conveyed by others' emotions. As mentioned previously, the relation between emotion recognition and expressiveness in PD patients has been documented a number of times [8, 10, 15]. Similar correlations in healthy participants have been shown by Künecke et al. [54], during a psychometric study in which participants had to choose which of the six basic emotions was shown in a video while having facial muscle activity measured through electromyography (EMG). Participants who demonstrated less EMG activity during the task also demonstrated worse emotion recognition, with a correlation coefficient of *r* = 0.32 between corrugator supercilii mimicry activity and anger recognition.

### 4.1. Embodied Simulation Theory

Embodied simulation theory suggests that facial mimicry aids in the recognition of emotions through a sensorimotor feedback loop [20, 21]. This theory is primarily supported by research investigating the consequences of blocking facial muscles during the observation of facial emotions. Oberman et al. [22] prevented participants from engaging in facial mimicry by having them either chew gum or bite a pen while engaging in an emotion recognition task. The pen-biters performed significantly worse at recognizing happiness than gum-chewers or control participants, presumably because the zygomaticus major muscles were tonically engaged. A similar methodology was employed by Ponari et al. [23], who used chopstick biting to demonstrate reductions in happiness and disgust recognition. These authors also used an eyebrow sticker-touching task to demonstrate reduction in anger recognition.

Chemical interference with muscular activation has also shown to impede emotion recognition. Neal and Chartrand [55] tested individuals with either botulinum toxin (Botox; which prevents muscle contraction) or dermal filler (Restylane; which leaves muscle activity intact) injections on the *Reading the Mind in the Eyes* task. This task presents participants with photographs of the eye region of the face during a particular expression and asks them to indicate the emotion expressed through the eyes. The Botox group performed worse at recognizing positive and negative expressions, regardless of response time. Importantly, these injections had been placed along the glabella (between eyebrows), forehead, and crow's feet (lateral sides of eyes), which reduced the ability to use surrounding facial muscles which are highly involved in emotional expression (e.g., corrugator supercilii and orbicularis oculi). Similarly, a study conducted by Hennenlotter et al. [56] established that interference with the corrugator supercilii muscles via Botox injection reduces brain activity in the left amygdala during imitation of angry facial expressions. Therefore, the amygdala might rely on facial feedback to process emotions (particularly anger). Overall, the results of these studies suggest that a reduction in the ability to express emotions creates changes in behaviour and brain activity related to emotional processing. For PD patients, deficits in emotional recognition could relate to the previously established reduction in expressiveness.

### 4.2. Neural Correlates of Facial Mimicry

A handful of studies have provided evidence that facial mimicry processes implicate areas of the brain which are affected by PD, including the basal ganglia and the mirroring system. Likowski et al. [25] made participants to passively view a series of stimuli depicting happy, sad, and angry expressions. Meanwhile, the activity of the zygomaticus major and corrugator supercilii muscles was recorded using EMG, and brain activity was recorded with fMRI. A regression analysis revealed that increased zygomaticus activity during happy faces correlated with activity in the caudate, an area of the basal ganglia that has been consistently associated with emotional activation [57, 58]. The caudate nucleus is implicated in reinforcement of goal-directed actions [59] hinting at the possibility that caudate's role in facial mimicry could be to mitigate the rewarding outcome of accurate emotion recognition. Furthermore, other research has found that an increase in mutual trust, but not distrust, during a social exchange activates the caudate, suggesting that this area of the basal ganglia is particularly involved in positive social encounters [60]. To corroborate this, Sims et al. [61] demonstrated that healthy participants mimic happy faces, but not angry faces, to a greater degree when the faces were previously conditioned to indicate a greater reward.

Rymarczyk et al. [26] corroborated Likowski et al.'s [25] results by demonstrating that the caudate was activated during facial mimicry of happy expressions but introduced the new finding that it might also mediate processing of angry expressions. Although caudate activity was not related to anger in Likowski et al.'s findings, this area has indeed been related to anger recognition [62] and avoidance behaviour [63]. In PD, the caudate receives fewer dopaminergic signals from the substantia nigra compared to controls and even has been shown to undergo morphological changes such as atrophy [64] and an altered shape [65]. Therefore, reduction of caudate activity in PD might underlie impairment of mimicry and/or its rewarding effects, producing deficits in emotion recognition.

Rymarczyk et al. [26] also revealed coactivation between the zygomaticus major and orbicularis oculi muscles during happy expressions and activity in the putamen, nucleus accumbens, and the globus pallidus, demonstrating that areas other than the caudate within the basal ganglia system are related to facial mimicry. These areas are often associated with feelings of positive affect (e.g., nucleus accumbens) [66] and have been shown to be active during expressions of happiness (e.g., putamen) [67]. Both of these areas are also impacted by PD and dopaminergic treatment [59, 68].

Upon viewing another individual's actions, the mirror neuron system in humans activates areas in the observer's brain that are associated with performing that action [69]. Neuroanatomically, it involves the inferior frontal operculum (IFO) and the inferior parietal lobule (IPL) [29,69–71]. Since facial mimicry is a mirroring behaviour, it is not surprising that these areas have been shown to be active during facial mimicry, particularly for happiness [25, 26, 72, 73]. Furthermore, Pohl et al. [29] demonstrated that these areas might be impacted by PD. PD patients and healthy controls completed the *Emotion Hexagon* task, which involves determining which proportion of particular emotions are shown in a photo. Brain activity was recorded with fMRI during this task. The authors demonstrated that controls had greater activity in the dorsal portion of the IFO and in the IPL compared to patients. Furthermore, for PD patients, activity in the IPL correlated with emotion recognition ability which corroborates the idea that mimicry and recognition are closely linked. The authors contend that reduced facial expressiveness due to motor disturbances as well as reduced activity in the IFO/IPL results in emotion recognition impairment for PD patients [29].

### 4.3. Facial Mimicry in Parkinson's Disease

The evidence reviewed so far has suggested that the impairments in emotional expressivity and recognition in PD patients might be linked by a reduction in facial mimicry. Neuroimaging studies seem to suggest that the areas involved in facial mimicry are commonly affected in PD. The notion that facial mimicry deficits contribute in some way to emotion recognition impairment in PD seems plausible. Surprisingly, there are currently only two studies investigating facial mimicry in PD patients [30, 31].

Livingstone et al. [31] recruited healthy controls and patients with mild-moderate PD (mean Hoehn and Yahr stage = 2.3) who were nondepressed, nondemented, and currently taking dopaminergic replacement therapy. The patients were tested while taking their usual dosage of prescribed medication. Both patients and controls had facial EMG activity of the left zygomaticus major, left corrugator supercilii, and right medial frontalis, recorded while being tested on a forced-choice emotion recognition task. The task used video stimuli of male and female actors depicting a particular facial expression while speaking or singing emotionally neutral phrases, and participants were asked to indicate whether the actor was calm, happy, sad, angry, fearful, surprised, disgusted, or neutral. Throughout the task, PD patients demonstrated greatly reduced mimicry to happy faces in the zygomaticus major muscles and to sad faces in the medial frontalis compared to controls. These effects were negatively correlated with behavioural response times. In other words, patients who displayed greater mimicry of emotions had shorter response times when identifying the emotional stimulus. Interestingly, although the mimicry response to anger in the corrugator supercilii muscles was slightly attenuated in PD patients compared to controls, there was no statistically significant difference between the groups for this particular emotion. Taken together, the results from Livingstone et al. [31] seem to suggest that there is a specific impairment in the mimicry of happiness and sadness in PD patients and that this impairment has behavioural consequences.

Argaud et al. [30] also recruited healthy controls and PD patients with mild PD (mean Hoehn and Yahr stage = 1.7) and an absence of depression, dementia, drug abuse, or other psychiatric illnesses. Patients were all taking dopaminergic replacement therapy and underwent testing on their usual dosage of medication. Patients and controls had muscle activity recorded via EMG at the left zygomaticus major, orbicularis oculi, and corrugator supercilii muscles while observing dynamic stimuli depicting happy, angry, and neutral emotions. After each avatar display, participants were asked to rate the degree of depicted emotional expression along seven visual analogue scales (VAS) corresponding to joy, sadness, fear, anger, disgust, surprise, and neutral. Several significant differences in emotion recognition and facial mimicry were observed. First, the PD patients had significantly lower accuracy scores for the VAS ratings compared to controls, particularly for happy and neutral expressions but not angry. Second, the PD patients demonstrated reduced corrugator supercilii mimicry to angry expressions compared to controls, and almost nonexistent zygomaticus major and orbicularis oculi mimicry toward happy faces. These results support those obtained by Livingstone et al. [31], suggesting that PD patients demonstrate reduced facial mimicry compared to controls, with a particular deficit in happy mimicry, which is related to problems identifying emotions.

Once again, based on the evidence synthesized in the present review, it is surprising that very few studies have investigated facial mimicry in PD patients. Many questions still remain unanswered, including whether dopaminergic treatment has an effect on facial mimicry, how the deficit is affected by the time course of the disease, and which brain structures underlie the reduction in mimicry. It is also interesting to note that both of these facial mimicry studies found deficits with happy expressions specifically, while performance with angry expressions was relatively spared. This is in contrast with the majority of emotion recognition studies in PD patients, which generally finds that happy face recognition remains high in PD patients [6, 8].

### 4.4. Compensatory Strategies

Studies of other clinical populations with facial movement dysfunction have shown a preserved ability to recognize emotions despite the inability to mimic. For example, a small sample of patients with Möebius syndrome (congenital facial paralysis) showed no impairment on a recognition test of the six basic emotions [74]. Interestingly, this preserved ability might be due to the development of compensatory mechanisms which facilitate emotion recognition in the absence of mimicry. Indeed, Goldman and Sripada [20] describe an alternate emotion simulation route that bypasses motor activation, going from the observation of an expression straight to neural representation and recognition of the emotion. Amazingly, there might be evidence that some PD patients are able to form compensatory mechanisms similar to those with Möebius syndrome in order to preserve emotion recognition when facial mimicry is reduced. In other words, PD patients might be able to become more reliant on some alternative route of emotion recognition. Wabnegger and colleagues [75] conducted an fMRI study comparing PD patients and healthy controls during negative emotion recognition (i.e., anger, fear, and sadness). The authors observed no differences between these two groups in their ability to recognize emotions or in the activated areas of the brain, except for greater activation of the somatosensory cortex in PD patients. Furthermore, the level of somatosensory cortex activation was positively correlated with emotion recognition ability. Similarly, Anders and colleagues [76] found increased activity in the right ventrolateral premotor cortex in asymptomatic *Parkin* mutation carriers compared to controls, which was positively correlated with emotion recognition ability. Again, this may demonstrate a compensatory mechanism involving movement preparation and sensory feedback signals which facilitates recognition when mimicry pathways are disrupted.

## 5. Conclusions

In this review, we have presented an argument suggesting that emotion recognition impairment in PD patients might be related to deficits in emotion expressivity. This could be due to hypomimia (i.e., hypokinesia and bradykinesia of facial expressions), cognitive impairment related to basal ganglia dysfunction, or possible dysfunction of the mirror neuron system. In sum, it is likely that PD patients demonstrate reduced facial mimicry which contributes to emotion recognition impairment. However, only two studies to date have examined facial mimicry abilities in PD patients, both of which have shown evidence of a deficit in facial mimicry.

An alternative explanation to the evidence presented in this review could argue that PD patients' reduction in facial mimicry is due to an initial inability to recognize the emotion to be mimicked, not the other way around. However, theorists hold that facial mimicry occurs prior to the recognition of an emotion for two main reasons, outlined by Goldman and Sripada [20]. First, facial mimicry is known to be a part of a more general mirroring system, which includes mimicry of more distal parts of the body (e.g., mimicking another person's leg crossing) which are not dependent on emotional processes. Although no studies have directly studied bodily mirroring behaviour in the PD population, the symptoms of bradykinesia and hypokinesia and evidence of a dysfunctional mirroring system in PD patients would suggest that mirroring behaviour is reduced overall in PD patients. Second, when facial muscles are manipulated into an expression without association with an emotion (e.g., when asked to say “cheese” to produce a smile), evidence shows that affective changes still ensue and that recognition of congruent emotions improves [77].

Embodied simulation theory strongly holds that facial mimicry precedes and aids in the recognition of emotions. Again, if mimicry is indeed reduced in PD patients, it is highly likely that this contributes to the symptom of impaired emotional recognition. Although only two studies have investigated facial mimicry in PD patients, both demonstrated a deficit in mimicry (particularly for happiness) which was coupled with behavioural consequences. There is a desperate need for more research into this area to determine the mechanisms behind mimicry and recognition deficits in PD patients. This enhanced understanding could lead to improved emotion processing and quality of life in PD patients.
